# An Improved Chimp-Inspired Optimization Algorithm for Large-Scale Spherical Vehicle Routing Problem with Time Windows

**DOI:** 10.3390/biomimetics7040241

**Published:** 2022-12-15

**Authors:** Yifei Xiang, Yongquan Zhou, Huajuan Huang, Qifang Luo

**Affiliations:** 1 College of Artificial Intelligence, Guangxi University for Nationalities, Nanning 530006, China; 2 Guangxi Key Laboratories of Hybrid Computation and IC Design Analysis, Nanning 530006, China

**Keywords:** chimp optimization algorithm, improved chimp optimization algorithm, spherical VRPTW model, metaheuristic algorithm

## Abstract

The vehicle routing problem with time windows (VRPTW) is a classical optimization problem. There have been many related studies in recent years. At present, many studies have generally analyzed this problem on the two-dimensional plane, and few studies have explored it on spherical surfaces. In order to carry out research related to the distribution of goods by unmanned vehicles and unmanned aerial vehicles, this study carries out research based on the situation of a three-dimensional sphere and proposes a three-dimensional spherical VRPTW model. All of the customer nodes in this problem were mapped to the three-dimensional sphere. The chimp optimization algorithm is an excellent intelligent optimization algorithm proposed recently, which has been successfully applied to solve various practical problems and has achieved good results. The chimp optimization algorithm (ChOA) is characterized by its excellent ability to balance exploration and exploitation in the optimization process so that the algorithm can search the solution space adaptively, which is closely related to its outstanding adaptive factors. However, the performance of the chimp optimization algorithm in solving discrete optimization problems still needs to be improved. Firstly, the convergence speed of the algorithm is fast at first, but it becomes slower and slower as the number of iterations increases. Therefore, this paper introduces the multiple-population strategy, genetic operators, and local search methods into the algorithm to improve its overall exploration ability and convergence speed so that the algorithm can quickly find solutions with higher accuracy. Secondly, the algorithm is not suitable for discrete problems. In conclusion, this paper proposes an improved chimp optimization algorithm (MG-ChOA) and applies it to solve the spherical VRPTW model. Finally, this paper analyzes the performance of this algorithm in a multi-dimensional way by comparing it with many excellent algorithms available at present. The experimental result shows that the proposed algorithm is effective and superior in solving the discrete problem of spherical VRPTW, and its performance is superior to that of other algorithms.

## 1. Introduction

The vehicle routing problem (VRP) is a famous path-planning problem that was first proposed by Dantzig and Ramser [1]. It has been widely studied in the field of optimization problems and is a very practical model (Toth et al. [2]). In recent years, many variants of the VRP have been developed, such as the VRP with time windows (VRPTW) (Yu et al. [3]), green VRP (GVRP) (Xu et al. [4]), capacitated VRP (CVRP) (Zhang et al. [5]), multi-depot VRP (MDVRP) (Duan et al. [6]), and heterogeneous VRP (HVRP) (Ghannadpour et al. [7]). To sum up, there are mainly three kinds of classical algorithms to solve VRPs, which are exact algorithms, traditional heuristic algorithms, approximation algorithms, and metaheuristic algorithms. Exact algorithms (such as branch-and-price (Li et al. [8]), sophisticated branch-cut-and-price methods (Pessoa et al. [9]), mixed-integer nonlinear programming algorithms (Xiao et al. [10]), approximate dynamic programming algorithms (Çimen et al. [11], etc.) use mathematical methods to search for the optimal solution. Although they can often find a good solution, there are also problems, such as the single form of the solution, inability to avoid exponential explosion, and consumption of a lot of computing time. The basic idea of traditional heuristic algorithms (such as saving algorithms (Li et al. [8]), improved Dijkstra algorithms (Behnk et al. [12]), etc.) is to start from the current solution, search for a better solution in its neighborhood to replace the current solution, and then continue to search until there are no better solutions (Da Costa et al. [13]). The traditional heuristic algorithm easily falls into the local optimum and cannot easily achieve the global optimum. In addition, approximation algorithms (Das et al. [14], Khachay et al. [15]) with theoretical performance guarantees and approximation schemes have been widely used to solve the problems of covering non-Euclidean settings. Metaheuristic algorithms have excellent performance and constantly perturb near the current solution to search for better solutions. Therefore, this study uses the meta-heuristic algorithm to solve the problem.

In recent years, many meta-heuristic algorithms have been proposed, and they are mainly divided into three categories: evolution-based algorithms (Back et al. [16]), physics-based algorithms (Webster et al. [17]), and swarm intelligence-based algorithms (Beni et al. [18]). The algorithm based on the evolution principle simulates the evolution of organisms. This method uses the crossover, mutation, and selection operators to update the population after randomly initializing the population and then continues to search for better solutions. The classical evolution-based algorithms mainly include the differential evolution algorithm (DE) (Storn [19]), genetic algorithm (GA) (Holland [20]), and biogeography-based optimization (BBO) (Simon [21]). The algorithm based on physical laws is inspired by physics and its individuals explore the solution space according to the physical law. The classical algorithms mainly include the black hole (BH) algorithm (Hatamlou [22]), gravity search algorithm (GSA) (Rashedi et al. [23]), big bang–big crash algorithm (BBBC) (Erol et al. [24]), and so on. The swarm intelligence algorithm mainly simulates the group behavior of organisms. Representative algorithms include the chimp optimization algorithm (Khishe et al. [25]), mayfly algorithm (Zervoudakis et al. [26]), equilibrium optimizer (Faramarzi et al. [27]), marine predator algorithm (Faramarzi et al. [28]), squirrel search algorithm (Jain et al. [29]), bald eagle search algorithm (Alsattar et al. [30]), Harris hawks optimization algorithm (Heidari et al. [31]), particle swarm optimization (Kennedy et al. [32]), artificial bee colony algorithm (Singh [33]), ant colony optimization algorithm (Neumann et al. [34]), firefly algorithm (Yang [35]), bat algorithm (Yang et al. [36]), grey wolf optimizer (Mirjalili et al. [37]), and the whale optimization algorithm (Mirjalili et al. [38]). Metaheuristic algorithms are widely used because of their excellent performance. Bi et al. (Bi et al. [39]) applied the GSTAEFA algorithm to the SMTSP. Artificial neural networks are widely used in image recognition and processing (Krizhevsky et al. [40]; Gu et al. [41]), time-series analysis (Arulkumaran et al. [42]; Tian et al. [43]), natural language processing (Juhn et al. [44]; Trappey et al. [45]), and building three-dimensional scenes (Dmitriev et al. [46]; Gkioxari et al. [47]). Genetic algorithms have been applied to engineering problems (Sayers [48]; Nicklow et al. [49]), classical optimization problems (Paul et al. [50]), and protein folding (Islam et al. [51]). The ant colony optimization algorithm has been applied to the traveling salesman problem (Dorigo et al. [52]; Dorigo [53]), engineering problems (Dorigo [53]; Nicklow et al. [49]), vehicle routing problems, machine learning, and bioinformatics problems (Dorigo et al. [52]), and the differential evolution algorithm has been used to solve the design problem of a reconfigurable antenna array. In addition, swarm intelligence algorithms have also been widely used in VRP. Solano Charris et al. [54] developed a local search metaheuristic algorithm to find the optimal path with the lowest cost in discrete scenarios. Wang et al. [55] used the multi-objective PSO algorithm to solve the dual objective model considering the time-varying speed of shared traffic resources. Chen et al. [56] proposed an intelligent water drop algorithm to solve the VRP of steel distribution. Zhang et al. [57] developed an improved tabu search algorithm to solve the cold chain logistics model. In GVRP, Zulvia et al. [58] solved the multi-objective model (GCVRPTW) using the multi-objective gradient evolution algorithm (MOGE).

However, many studies on the VRPTW are generally carried out on two-dimensional planes. In many research fields, the three-dimensional spherical structure is also of great significance. For example, celestial bodies, particle structures, daily foods, proteins and other nutrients, balls, buildings, and path-planning problems are all problems related to spheres. Therefore, it is of great practical significance to expand the research on the VRPTW from the two-dimensional plane to three-dimensional spheres. Zhang et al. [59] applied the BBMA to the spherical MST problem. To sum up, in order to carry out research related to the distribution of goods by unmanned vehicles and unmanned aerial vehicles, this paper plans the path of a robot through these coordinates in three-dimensional space in order to carry out research on the VRPTW in the spatial dimension.

ChOA has been applied to various practical problems, such as image segmentation for medical diagnosis (Si et al. [60]), clustering analysis (Sharma et al. [61]; Yang et al. [62]), Said–Ball curve degree optimization (Hu et al. [63]), and convolution neural networks (Chen et al. [64]). In addition, Du and Zhou (Du et al. [65]; Du et al. [66]) improved this algorithm and applied it to 3D path-planning problems and color image-enhancement problems. The algorithm divides the population of each generation into four groups, namely attackers, barriers, chasers, and drivers, and they cooperate against prey. Therefore, different groups search different spaces, which enhances the searching ability of ChOA. The adaptive factor of ChOA has a faster convergence speed and can adaptively balance exploration and exploitation, but it also easily falls into the local optimum. In order to obtain a group of better solutions with limited resources and time, this paper proposes an improved ChOA (MG-ChOA) for solving the spherical VRPTW model. The main contributions of this paper are as follows. Firstly, the proposed algorithm combines the ChOA algorithm with the quantum coding, local search, multiple population, and genetic operators to ensure that the algorithm can not only achieve adaptive and rapid convergence, but also find solutions with higher accuracy. Secondly, this paper proposes a three-dimensional spherical VRPTW and applies the proposed algorithm to solve this problem. Finally, by comparing with the running result of popular swarm intelligence algorithms for eight different instances, the effectiveness and superiority of the proposed algorithm in dealing with large-scale combinatorial optimization problems are strongly verified.

The remaining organizational structure of this paper is as follows. Section 2, the Related Work, briefly depicts works related to the model proposed. Section 3 analyzes two-dimensional VRPTW and spherical VRPTW models to propose the mathematical model of a spherical VRPTW. The proposed algorithm (MG-ChOA) for a spherical VRPTW, an improved MG-ChOA algorithm based on ChOA, is presented in Section 4. The discussion of the experimental results analyzes the performance of the algorithm in Section 5. The conclusion and future work proposals are presented in Section 6.

## 2. Related Work

### 2.1. Geometric Definition of Sphere

**Figure 1:**
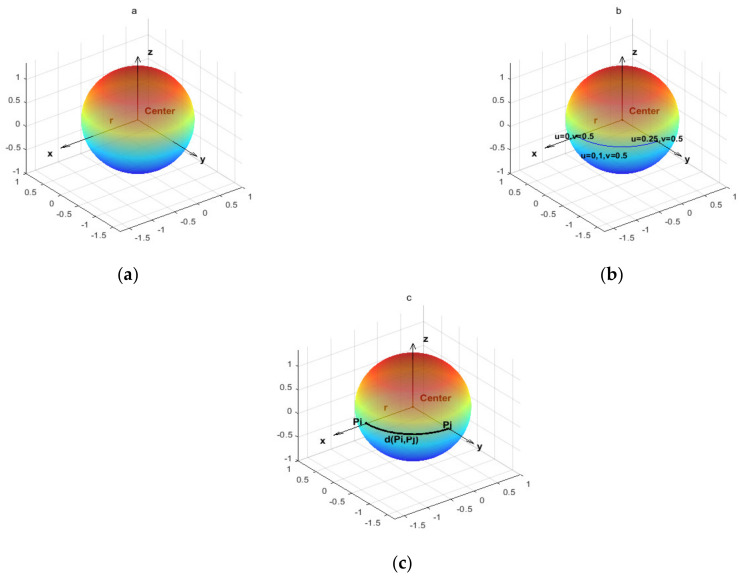
(**a**) represents the geometric definition of the sphere; (**b**) represents the definition of points on the sphere; (**c**) represents the geodesics between two points on the sphere, and the colors represents the height z.

A sphere refers to a set of points in 3D space with equal distance from the center point of the sphere, and radius is the distance from the center point of the sphere to a point on the sphere, as shown in Figure 1a. Therefore, a sphere with radius *r* can be defined by the following formula.

(1) $$x^{2}+y^{2}+z^{2}=r^{2}$$

where *x*, *y*, and *z* are coordinate axes of three-dimensional space, which are used to describe each point.

### 2.2. Definition of Points on the Sphere

The coordinates of points on the sphere can be described in detail with the following equation (Hearn et al. [67]).

(2) ps(u,v)=(x(u,v),y(u,v),z(u,v))

The coordinate of each point can be represented by *x*, *y*, and *z*, and they can be expressed by normalized parameters (such as *u* and *v*) in [0, 1]. Equations (3)–(5) specify the coordinates of each point on the sphere (Eldem et al. [68]).

(3) x(u,v)=rcos(2πu)sin(πu)

(4) y(u,v)=rsin(2πu)cos(πu)

(5)  z(u,v)=rcos(πu)

where *u* and *v* respectively represent the longitude and latitude lines used to calibrate the position, as shown in Figure 1b. Different combinations of *u* and *v* describe points of the sphere (Uğur et al. [69]), as shown in Figure 1b. In order to save computing resources and compare the performance of algorithms more conveniently, this study uses a sphere with a radius of one to carry out experiments and discussions.

### 2.3. Geodesics between Two Points on the Sphere

The big circle is a figure formed by the intersection of a plane passing through the sphere’s center. The shortest path between two points on the sphere is a certain arc length of the big circle, and the geodesic line is this arc (Lomnitz [70]). According to the description above, the geodesic line between $p_{i}$ (point *i*) and $p_{j}$ (point *j*) on the sphere is shown in Figure 1c, which can be described by vector $\vec{v_{i}}$ and vector $\vec{v_{j}}$, respectively, and their product is defined as follows.

(6) $$\vec{v_{i}}\vec{v_{j}}=\left|\vec{v_{i}}\right|\left|\vec{v_{j}}\right|cos\theta$$

Or

(7) $$\vec{v_{i}}\vec{v_{j}}=x_{i}x_{j}+y_{i}y_{j}+z_{i}z_{j}$$

where *θ* indicates the angle of two vectors, and the formula of the shortest path can be expressed as follows.

(8) $${\overset{\wedge }{d}}_{{}_{p_{i}p_{j}}}=r\theta$$

According to Formulae (6)–(8), we get

(9) $${\overset{\wedge }{d}}_{{}_{p_{i}p_{j}}}=r\;arccos(\frac{x_{i}x_{j}+y_{i}y_{j}+z_{i}z_{j}}{r^{2}})$$

Calculate the distance between two points on a sphere with *n* points to obtain a symmetric distance matrix *Dis* with the size of *n* × *n*, and the distance matrix can be described as follows.

(10) $$Dis=\left[\begin{array}{l} {\overset{\wedge }{d}}_{11}\;{\overset{\wedge }{d}}_{12}\;\cdots \;{\overset{\wedge }{d}}_{1n}\\ {\overset{\wedge }{d}}_{21}\;{\overset{\wedge }{d}}_{22}\;\cdots \;{\overset{\wedge }{d}}_{2n}\\ \;\vdots \;\vdots \;\ddots \;\vdots \\ {\overset{\wedge }{d}}_{n1}\;{\overset{\wedge }{d}}_{n2}\;\cdots \;{\overset{\wedge }{d}}_{nn} \end{array}\right]=\left[\begin{array}{l} \infty \;{\overset{\wedge }{d}}_{12}\;\cdots \;{\overset{\wedge }{d}}_{1n}\\ {\overset{\wedge }{d}}_{21}\;\infty \;\cdots \;{\overset{\wedge }{d}}_{2n}\\ \;\vdots \;\;\vdots \;\ddots \;\vdots \\ {\overset{\wedge }{d}}_{n1}\;{\overset{\wedge }{d}}_{n2}\;\cdots \;\infty \end{array}\right]$$

where ${\overset{\wedge }{d}}_{ij}$ denotes the distance of the geodesic line formed by two points on the sphere and ${\overset{\wedge }{d}}_{ij}$ equals infinity, meaning that the point cannot reach itself.

## 3. Mathematical Model of Spherical VRPTW

### 3.1. VRPTW on a 2D Plane

**Figure 2:**
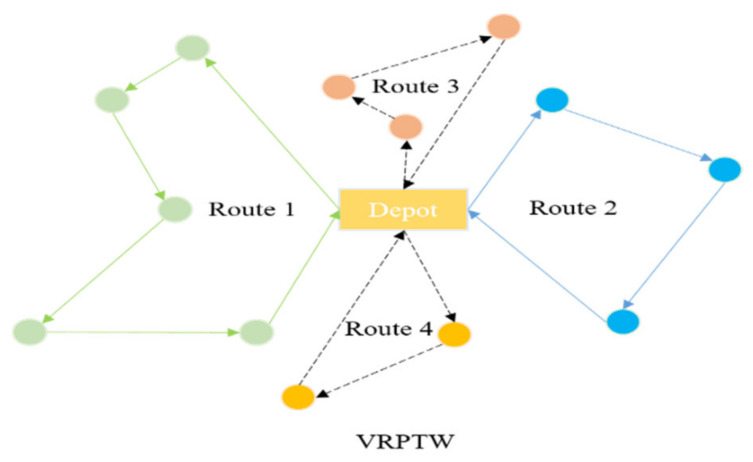
Routes of VRPTW, different colors denote the different routes, circles denotes the customers in these routes, and the arrows denotes the routes.

VRPTW describes a path-planning problem of allocating goods from a distribution center to different customers within a specified time, including *K* vehicles and *N* customer nodes. The logistics network reasonably plans a series of routes to serve customers according to the transportation demand, and vehicles must leave and finally return to the depot. As the loading capacity of each vehicle is limited, the transportation company must serve customers within a specified time to meet the customers’ needs. In addition, if the vehicle arrives at the customer node in advance, it needs to wait for a period of time before starting the service. Figure 2 shows the process of VRPTW. The parameters used in the model are shown in Table 1.

$\begin{array}{l} x_{ijk}=\left\{\begin{array}{l} 1,\mathrm{if}\;\mathrm{vehicle}\;k\;\mathrm{travels}\;\mathrm{from}\;i\;\mathrm{to}\;j;\\ 0,other. \end{array}\right.\\ y_{ik}=\left\{\begin{array}{l} 1,\mathrm{if}\;\mathrm{node}\;i\;\mathrm{is}\;\mathrm{served}\;\mathrm{by}\;\mathrm{vehicle}\;k;\\ 0,other. \end{array}\right. \end{array}$

Therefore, the dual-objective mathematical model of VRPTW can be expressed as follows:

(11) $$\mathrm{TD}=\mathrm{Min}\;\sum_{i\in C}\sum_{j\in C}\sum_{k\in V}d_{ij}x_{ijk}$$

(12) $$\mathrm{NV}=\mathrm{Min}\;\sum_{j\in C}\sum_{k\in V}x_{0jk}$$

Subject to:

(13) $$\sum_{i\in C}q_{i}y_{ik}\leq Q_{k},\;\forall k\in V$$

(14) $$\sum_{k\in V}y_{ik}=1,\;\forall i\in C$$

(15) $$\sum_{i\in C}x_{ijk}=y_{jk},\;\forall j\in C,\;\forall k\in V$$

(16) $$\sum_{j\in C}x_{ijk}=y_{ik},\;\forall i\in C,\;\forall k\in V$$

(17) $$\sum_{j\in C}x_{0jk}=\sum_{j\in C}x_{j0k}=1,\;\forall k\in V$$

(18) $$ET_{i}<T_{ik}<LT_{i},\;\forall i\in C$$

(19) $$ET_{i}<T_{ik}+w_{ik}+ST_{i}+t_{ij}<LT_{j},\;\forall i,j\in C,\;\forall k\in V$$

Equations (11) and (12) represent objective functions consisting of the travel distance and the number of vehicles. Equation (13) indicates that each customer’s demands on a route should not be greater than the maximum capacity of the vehicle. Equation (14) denotes that one customer should only be served once. Equation (15) indicates that the vehicle only serves one node before serving the next node. Equation (16) denotes that vehicles only visit one node after visiting the previous node. Equation (17) indicates that the start and end nodes of each vehicle should return to the depot. Equations (18) and (19) are time window constraints.

### 3.2. Three-Dimensional Spherical VRPTW Model

The 3D spherical VRPTW model maps the customer nodes from the 2D plane model to the 3D space. Therefore, each customer node of *i* can be defined as $c_{i}=(x_{i},y_{i},z_{i})$. Therefore, the mathematical model of the three-dimensional spherical VRPTW model can be defined as follows:

(20) $$\mathrm{TD}=\mathrm{Min}\;\sum_{i\in C}\sum_{j\in C}\sum_{k\in V}\overset{\wedge }{d_{ij}}x_{ijk}$$

In the formula above, $\overset{\wedge }{d_{ij}}$ and the symmetric matrix *Dis* can be calculated by Equations (9) and (10), respectively. Similarly, constraint conditions can be represented by Equations (13)–(19).

## 4. The Proposed Algorithm (MG-ChOA) for the Spherical VRPTW

### 4.1. The Chimp Optimization Algorithm (ChOA)

The ChOA was first proposed by Khishe and Mosavi [17] in 2020, and it simulates the predatory behavior of chimps. The algorithm divides the population of each generation into four groups, namely attackers, barriers, chasers, and drivers, and they cooperate against prey. The ChOA has excellent adaptive factors that can help it balance exploration and exploitation so as to find better solutions. The mathematical model of ChoA is as follows:

(21) $$\vec{d}=\left|\vec{c}{\vec{x}}_{prey}(t)-\vec{m}{\vec{x}}_{chimp}(t)\right|$$

(22) $${\vec{x}}_{chimp}\left(t+1\right)={\vec{x}}_{prey}\left(t\right)-\vec{a}\vec{d}$$

Equations (21) and (22) describe the chasing and driving processes of the algorithm. Among them, ${\vec{x}}_{chimp}$ and ${\vec{x}}_{prey}$ represent the coordinates of the individual and prey, respectively, and t represents the current number of iterations. In addition, $\vec{a}$, $\vec{c}$, and $\vec{m}$ represent coefficient vectors, which are determined by Equations (23)–(25), respectively.

(23) $$\vec{a}=2\vec{f}\vec{r_{1}}-\vec{f}$$

(24) $$\vec{c}=2\vec{r_{2}}$$

(25) $$\vec{m}=chaotic\_value$$

where $\vec{f}$ refers to a vector linearly decreasing in the interval of [2.5, 0], $\vec{r_{1}}$ and $\vec{r_{2}}$ represent random vectors that each dimension falls in the interval of [0, 1], and $\vec{m}$ represents a random vector obtained from chaotic mapping functions.

The model above describes the main flow of the algorithm. In each iteration, the algorithm firstly selects the four best individuals, and then the remaining individuals update their positions based on them. The specific mathematical model of the algorithm is as follows:

(26) $$\begin{array}{l} {\vec{d}}_{Attacker}=\left|{\vec{c}}_{1}{\vec{x}}_{Attacker}-{\vec{m}}_{1}\vec{x}\right|,\;{\vec{d}}_{Barrier}=\left|{\vec{c}}_{2}{\vec{x}}_{Barrier}-{\vec{m}}_{2}\vec{x}\right|\\ {\vec{d}}_{Chaser}=\left|{\vec{c}}_{3}{\vec{x}}_{Chaser}-{\vec{m}}_{3}\vec{x}\right|,\;{\vec{d}}_{Driver}=\left|{\vec{c}}_{4}{\vec{x}}_{Driver}-{\vec{m}}_{4}\vec{x}\right| \end{array}$$

(27) $$\begin{array}{l} {\vec{x}}_{1}={\vec{x}}_{Attacker}-{\vec{a}}_{1}{\vec{d}}_{Attacker},\;{\vec{x}}_{2}={\vec{x}}_{Barrier}-{\vec{a}}_{2}{\vec{d}}_{Barrier}\\ {\vec{x}}_{3}={\vec{x}}_{Chaser}-{\vec{a}}_{3}{\vec{d}}_{Chaser},\;{\vec{x}}_{4}={\vec{x}}_{Driver}-{\vec{a}}_{4}{\vec{d}}_{Driver} \end{array}$$

(28) $$\vec{x}(t+1)=\frac{{\vec{x}}_{1}+{\vec{x}}_{2}+{\vec{x}}_{3}+{\vec{x}}_{4}}{4}$$

The random vector $\vec{c}$ strengthens (c > 1) or weakens (c < 1) the moving range of prey. When the random vector $\vec{a}$ is greater than 1 or less than −1, the algorithm will be in the exploration stage; otherwise, it will be in the exploitation stage. Therefore, the algorithm can adaptively adjust exploration and exploitation to find a better solution. The pseudo code of ChOA is shown in Algorithm 1.
**Algorithm 1**: The pseudo code of ChOA1. Initialize the population ${\vec{x}}_{i}$(*i* = 1,…, *N*).2. Set *f, a, c, m* = *chaotic_value*, and *u* is a random number in [0, 1].3. Calculate individuals’ fitnesses.4. Select four leaders.5. **while**
*Iter*
**<**
*Max_iter*6.   **for** each individual7.    Update ***f****, **a**, **c**, m* based on Equations (23)–(25).8.   **end for**9.    **for** each agent10.    **if** (*u* < 0.5)11.      **if** (|***a***| < 1)12.    Update its position based on Equations (26)–(28).13.      **else if** (|***a***| > 1)14.    Select a random individual.15.       **end if**16.    **else if** (*u* > 0.5)17.    Update its position based on a *chaotic_value*.18.    **end if**19.   **end for**20.    Calculate individuals’ fitnesses and select four leaders.21.    ***t* = *t* +** 1.22. **end while**23. Obtain the best solution.

### 4.2. The Proposed MG-ChOA for the Spherical VRPTW

ChOA can adaptively adjust exploration and exploitation and its convergence speed is fast, but it still has the shortcomings of limited exploration capacity, easily falling into the local optimum, and not being suitable for discrete problems. Therefore, this paper improves the ChOA algorithm to solve the three-dimensional spherical VRPTW. The proposed algorithm (MG-ChOA) introduces the quantum coding method, multiple-population strategy, genetic operators, and local search strategy to improve the search ability of the algorithm. Using the quantum coding method to initialize the population can increase the population’s diversity at the initial stage. Similarly, the multiple-population strategy can increase the population diversity of the algorithm in the iterative process to find better solutions. Genetic operators enhance the exploration ability of the algorithm, and the local search strategy helps the algorithm to search for better solutions in the neighborhood of each solution.

#### 4.2.1. Encoding and Decoding of the Spherical VRPTW

**Figure 3:**
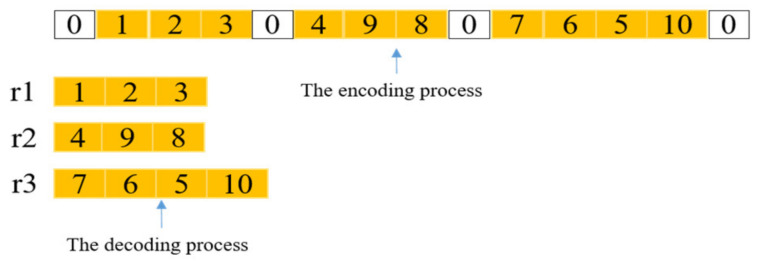
The encoding and decoding processes, 0 represents the depot, and other numbers denote customers.

As shown in Figure 3, each code is divided into many small parts by the number of 0 that represents the distribution center, and the remaining numbers represent customer nodes. Each independent small part above represents one path served by one vehicle. Therefore, the path encoding of Figure 3 can be sequentially decoded into sequences of [1, 2, 3], [4, 9, 8], and [7, 6, 5, 10].

#### 4.2.2. Initializing Population Using the Quantum Coding

In this study, we used quantum coding to initialize the population. This method enhances the population’s diversity at the beginning of the iteration, which is conducive to enabling the algorithm to quickly find a better solution at the initial stage. The smallest unit of quantum computing is the quantum bit (qubit), whose state is the superposition state of 0 or 1. The quantum bit is defined as follows.

(29) $$\left|\varphi \rangle =\alpha \right.\left|0\rangle +\beta \left|1\rangle \right.\right.$$

where α and β are complex numbers and $\left|\alpha^{2}\right|\;\mathrm{and}\;\left|\beta^{2}\right|$ respectively represent the probability of the state being 0 or 1, and they satisfy the equation of $\left|\alpha^{2}\right|+\left|\beta^{2}\right|=1$. The specific representation of a qubit is as follows:

(30) $$\left|\varphi \right.\rangle =\left[\begin{array}{c} \alpha \\ \beta \end{array}\right]=\left[\begin{array}{c} \cos(\theta )\\ \sin(\theta ) \end{array}\right],\theta \in [0,2\pi ]$$

Inspired by the quantum encoding, individuals of the population can be described as follows:

(31) $$QC_{i}=(\varphi_{1},\varphi_{2},\ldots \varphi_{d})=\left(\begin{array}{c} \cos(\theta_{i1}),\cos(\theta_{i2}),\ldots \cos(\theta_{id})\\ \sin(\theta_{i1}),\sin(\theta_{i2}),\ldots \sin(\theta_{id}) \end{array}\right)$$

where $QC_{i}$ denotes the ith individual of the population, θ denotes the angle falling in the interval of [0, 2π], and n and d represent the total number and dimensions of each individual, respectively. Therefore, each individual has two candidate schemes, which can be defined as follows:

(32) $$\begin{array}{l} QC_{ic}=\left(\cos(\theta_{i1}),\cos(\theta_{i2}),\ldots \cos(\theta_{id})\right)\\ QC_{is}=\left(\sin(\theta_{i1}),\sin(\theta_{i2}),\ldots \sin(\theta_{id})\right) \end{array}$$

The initialization population of the proposed algorithm consists of n individuals and d dimensions, so the initialization population is to construct a matrix of n × d. According to the rules of quantum coding, we firstly needed to initialize the angle matrix and then obtain the initialization population according to the angle matrix. The angle matrix can be calculated by the following formula:

(33) $$\theta_{ij}=lb_{ij}+rand(0,1)●(ub_{ij}-lb_{ij}),\;1\leq i\leq n,\;1\leq j\leq d$$

where $lb_{ij}$ and $ub_{ij}$ respectively represent the minimum and maximum values of each of the individuals’ dimensions at the problem boundary, and their values are 0 and 2 π, respectively, while rand indicates random numbers falling in the interval of [0, 1]. The initialized angle matrix is as follows.

(34) $$\theta =\left[\begin{array}{l} \theta_{11}\;\theta_{12}\;\cdots \;\theta_{1d}\\ \theta_{21}\;\theta_{22}\;\cdots \;\theta_{2d}\\ \;\vdots \;\vdots \;\ddots \;\vdots \\ \theta_{n1}\;\theta_{n2}\;\cdots \;\theta_{nd} \end{array}\right]$$

where *QC* represents a population of N individuals. Each individual has two positions in the space, and they represent the candidate solution of the problem. Therefore, each individual has two different candidate solutions. To sum up, the quantum population can be defined by the following formula.

(35) $$QC=\left[\begin{array}{l} QC_{1}\\ QC_{2}\\ \;\;\;\vdots \;\\ QC_{n} \end{array}\right]=\left[\begin{array}{l} QC_{1c}\\ QC_{1s}\\ QC_{2c}\\ QC_{2s}\\ \;\;\;\vdots \\ QC_{nc}\\ QC_{ns} \end{array}\right]=\left[\begin{array}{l} \cos(\theta_{11}),\cos(\theta_{12}),\ldots \cos(\theta_{1d})\\ \sin(\theta_{11}),\sin(\theta_{12}),\ldots \sin(\theta_{1d})\\ \cos(\theta_{21}),\cos(\theta_{22}),\ldots \cos(\theta_{2d})\\ \sin(\theta_{21}),\sin(\theta_{22}),\ldots \sin(\theta_{2d})\\ \;\;\;\;\;\vdots \;\\ \cos(\theta_{n1}),\cos(\theta_{n2}),\ldots \cos(\theta_{nd})\\ \sin(\theta_{n1}),\sin(\theta_{n2}),\ldots \sin(\theta_{nd}) \end{array}\right]$$

According to Equation (35), the distribution of individuals in the initial population is closely related to two different trigonometric functions. The value distribution of sine and cosine functions greatly affects the distribution of the population, which can not only enhance the diversity of the population distribution in the solution space, but also balance the initial exploration and exploitation; therefore, this is helpful for the algorithm to find excellent solutions quickly. In addition, individuals obtained by the formula above are all decimals in the interval of [0, 1]. Therefore, the proposed algorithm converts these decimals into percentages and then multiplies them by the total number of customers, rounds them, and obtains the route code. Finally, we removed and reinserted the duplicated part of the route code to obtain the final initialization population.

#### 4.2.3. The Multiple-Population Strategy for MG-ChOA

**Figure 4:**
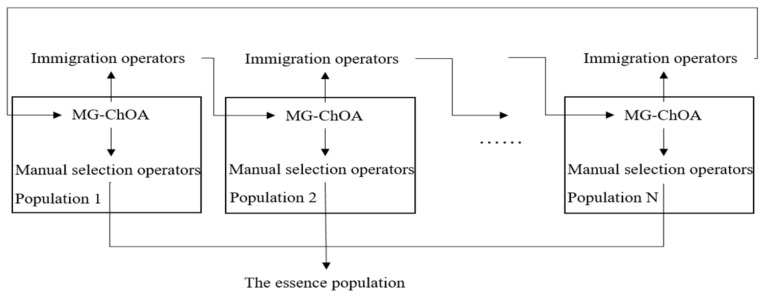
The description of the multiple-population strategy.

The main method of the multiple-population search strategy is to use multiple populations with different parameters to search the target solution together, and it mainly includes the immigration and manual selection operator. When the algorithm iteratively searches for the target solution, the migration operator is used to contact and exchange the optimal solution among various populations, and the optimal solution of each generation is saved to the essence population by the manual selection operator as the basis of algorithm convergence. The whole process above is shown in Figure 4. As shown in the figure, when the population 1-N searches the solution space, the migration operator will replace the worst individual of population i + 1 with the best individual of population i, and the best solution of population N will be used to replace the worst solution of the first population. Therefore, information exchange between populations is achieved.

#### 4.2.4. Genetic Operators

**Figure 5:**
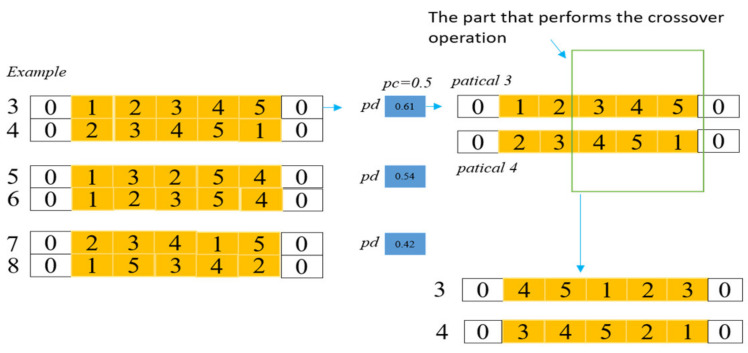
The mechanism of the genetic operators, 0 represents the depot, other numbers denote customers, and blue marks denote different probabilities.

The proposed algorithm introduces genetic operators to help the algorithm update the population to search for excellent individuals in the solution space. Specifically, the proposed algorithm firstly selects some excellent individuals as a new population, then assigns a probability pd to each individual in the new population; if it is greater than or equal to 0.5, the current individual will cross with the next individual. Figure 5 shows an example of the crossover operation, in which the numbers 3–8 represent the selected individuals, and then the algorithm assigns a random value pd to each of them. From the method above, pd of 3 and 5 are numbers greater than 0.5, so they were selected to cross with their next individual. Moreover, mutation is also an integral part of genetic operators. Similarly, the proposed algorithm assigns a probability pmd to each individual in the population, and if it is greater than the mutation probability pm given in this paper, the individual will perform the mutation operation. Therefore, a new route can be obtained by exchanging randomly selected customers on two individuals. The introduction of genetic operators enhances the exploration ability of the proposed algorithm.

#### 4.2.5. Local Search Strategy

**Figure 6:**
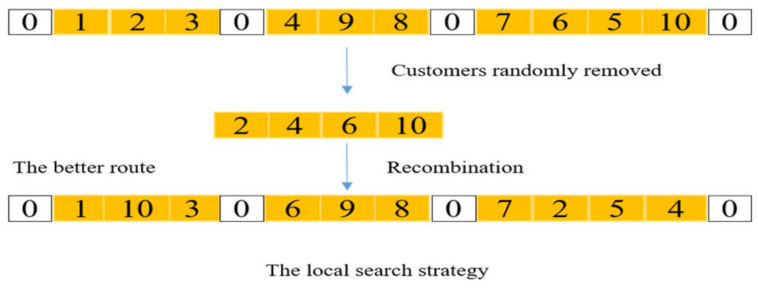
The local search method, 0 represents the depot and other numbers denote customers.

The local search strategy is helpful for the proposed method to search in the neighborhood of a specific solution to find a better solution. This method allows customers to move on the path in a certain way, and finally obtains more potential individuals without violating various constraints. The algorithm proposed in this paper uses a mechanism to generate a number of customers to be removed in the search process, and then reinserts the removed customers in a more reasonable location without violating the constraints of the time window and capacity. Therefore, better solutions in the neighborhood will be obtained. Figure 6 shows that a better route was obtained by randomly removing some customers and reinserting them in a more appropriate location.

#### 4.2.6. Computational Complexity Analysis

**Figure 7:**
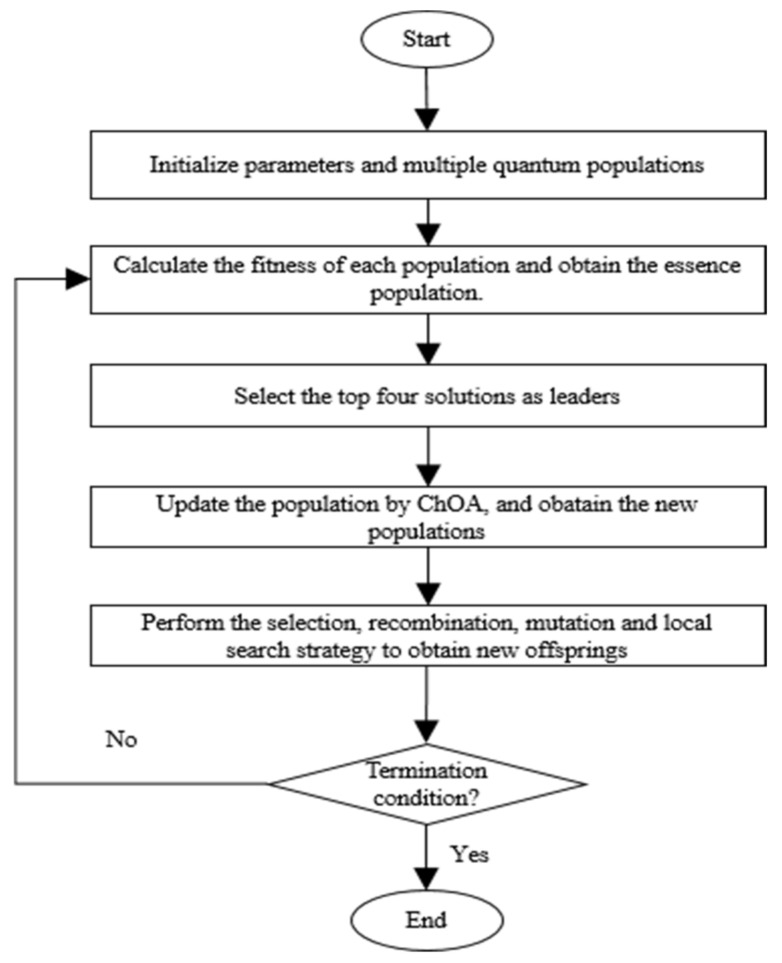
The flowchart of the proposed MG-ChOA algorithm.

The complexity of the initialization stage is O (C × N × d), where C represents the number of populations, N and d respectively represent the number of individuals in each population and the dimensions of each individual. The complexity of calculating fitness is O (T × C × N), where T indicates the number of iterations. The complexity of the essence population extraction is O (C), and the complexity of updating the population through ChOA is the same as that through genetic operators and local search, which is O (T × C × N × d). In general, the computational complexity of this method is O (T × C × N × d), and the pseudo code and flow chart of the proposed algorithm in this paper are shown in Algorithm 2 and Figure 7, respectively.
**Algorithm 2**: The pseudo code of the proposed MG-ChOA algorithm1. Initialize **f**, **a**, **c**, **m**, the probability of crossover and mutation. 2. Initialize multiple quantum populations,$x_{i}$ (i = 1,…, N). 3. **while** Iter < Max_iter4.    Calculate the fitness of each population and obtain the essence population.5.    Select the top four solutions from the essence population as leaders. 6.    Update each population by ChOA, and obtain new populations.7.    Perform the selection, recombination, mutation, and local search strategy to obtain the offspring.8.    Update **f**, **a**, **c, m** based on Equations (23)–(25).9. **end while**10. Obtain the optimal individual and accomplish data saving.

## 5. Experimental Results and Discussion

This paper uses eight different cases to test MG-ChOA’s ability to solve spherical VRPTW. All experiments were conducted on the premise that r equals one, and the numbers of customer nodes in these instances were 80, 100, 200, 400, 600, 800, 1000, and 1200. For cases with fewer than 100 customers, the time window and load data were from the Solomon dataset (Solomon [71]), and these data of the remaining cases were generated randomly. Owing to the strong randomness of metaheuristic algorithms, all algorithms were independently run 30 times in each case to obtain the result. The structure of this chapter is as follows. Firstly, Experimental Setup provides the parameter settings of all algorithms and experimental configurations. Secondly, this paper presents the results of the proposed algorithm running for 2D instances. Moreover, this paper compares the search ability and results of the proposed algorithm with other algorithms in low- and high-dimensional cases. Finally, this paper discusses the impact of different improvement methods on the overall performance of the algorithm.

### 5.1. Experimental Setup

The code of all experiments carried out in this paper was compiled in MATLAB. The computer system was configured with an Intel Core Intel (R) Core (TM) i7-9700 CPU, 16 GB RAM, and Windows 10 operating system. In this experiment, each algorithm iterated 300 generations and the population size of all algorithms was set to 100. This paper also compares the performance of MG-ChOA with the GA, ant colony algorithm (ACO), PSO, slime mold algorithm (SMA), firefly algorithm (FA), chimp optimization algorithm (ChOA), and gray wolf optimizer (GWO). The GA was integrated with the local search strategy. Moreover, many excellent improved algorithms (RPSO (Borowska [72]), JADE (Su et al. [73]), L-SHADE (Chen et al. [74]), learning CSO (Borowska [75]), and CMA-ES (Tong et al. [76])) should also be used to comprehensively analyze the performance of the proposed algorithm, and this study selected two of them (RPSO, JADE). In order to verify the effectiveness and superiority of the MG-ChOA algorithm, this paper also comprehensively compares the convergence curve, ANOVA test, fitness value obtained by 30 runs, Wilcoxon rank-sum test (Gibbons et al. [77]; Derrac et al. [78]), effects of different improvement methods, and running results. In addition, the control parameter for each algorithm was set as follows (Table 2) (Zhang et al. [59]).

### 5.2. Performance Comparison of Algorithms for Two-Dimensional Datasets

**Figure 8:**
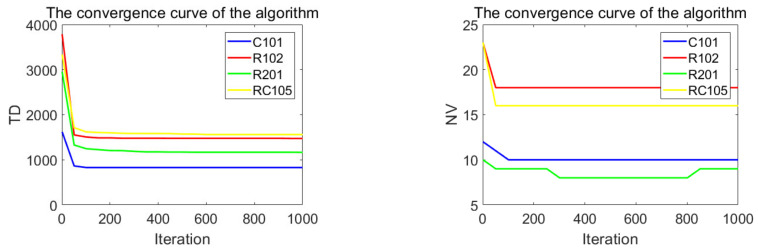
The convergence curve after 1000 iterations of the algorithm.

**Figure 9:**
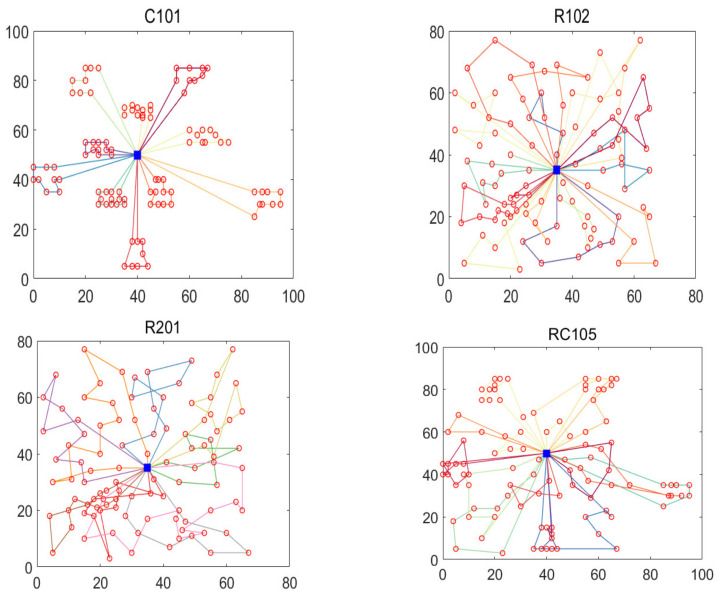
The optimal routes for four datasets, different colored lines denote the different routes, and the red circles represent the customers.

In order to analyze the performance of the proposed algorithm in multiple dimensions, this chapter briefly analyzes the results of the proposed algorithm in the two-dimensional plane. This research selected four different Solomon datasets to test the algorithm, which were C101, R102, R201, and RC105. The differences between the algorithm and the most famous results are provided in Table 3. Figure 8 shows the convergence speed of this algorithm. Figure 9 shows the optimal path obtained by the algorithm for each dataset. Table 3 shows that the performance of the algorithm for four different datasets was significantly different from the most famous results, with minimum and maximum values of 0 and 7%. Figure 8 shows the superior convergence performance of the algorithm, which converged to the optimal value quickly for all datasets. In conclusion, the results of four 2D datasets show the effectiveness of the proposed algorithm.

### 5.3. Performance Comparison of Algorithms for Low-Dimensional Instances

**Figure 10:**
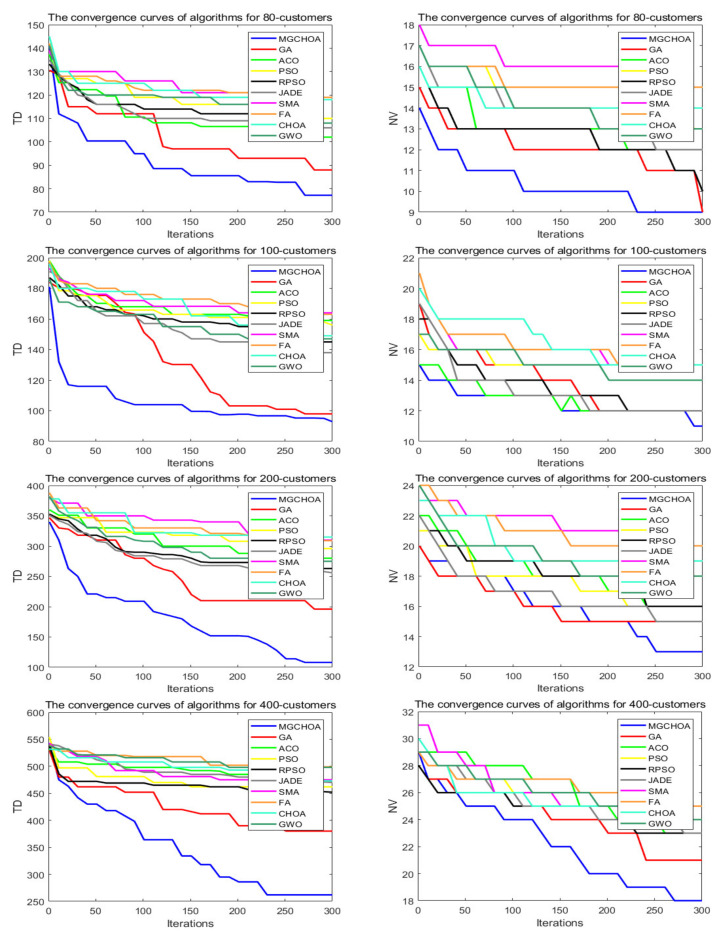
The convergence curves after 300 iterations of algorithms.

**Figure 11:**
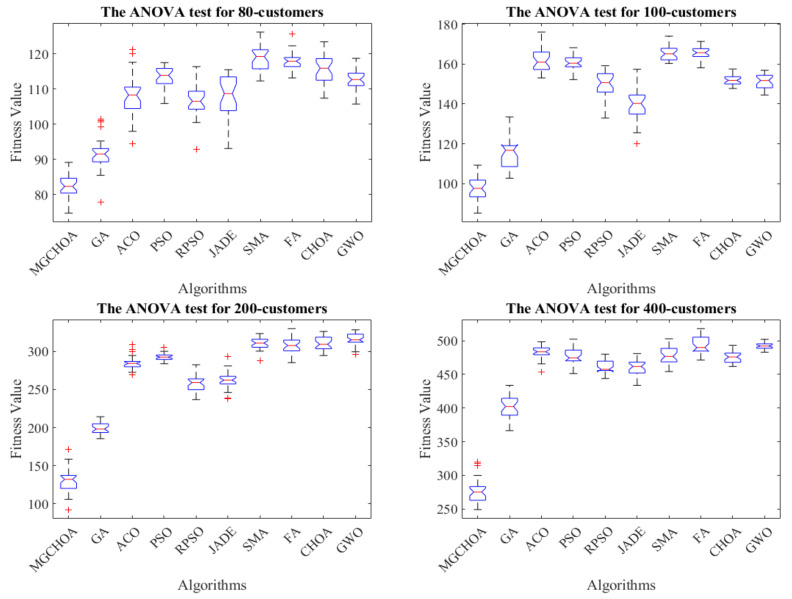
The ANOVA tests of the results after 30 runs, the blue box denotes the data distribution, the red line represents the median value, and the red symbol denotes the abnormal value.

**Figure 12:**
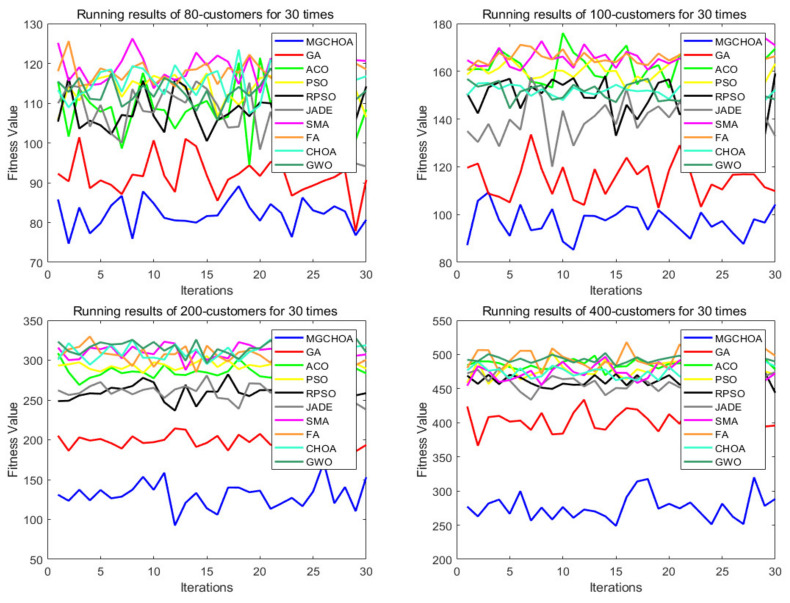
The results of 30 runs by algorithms.

**Figure 13:**
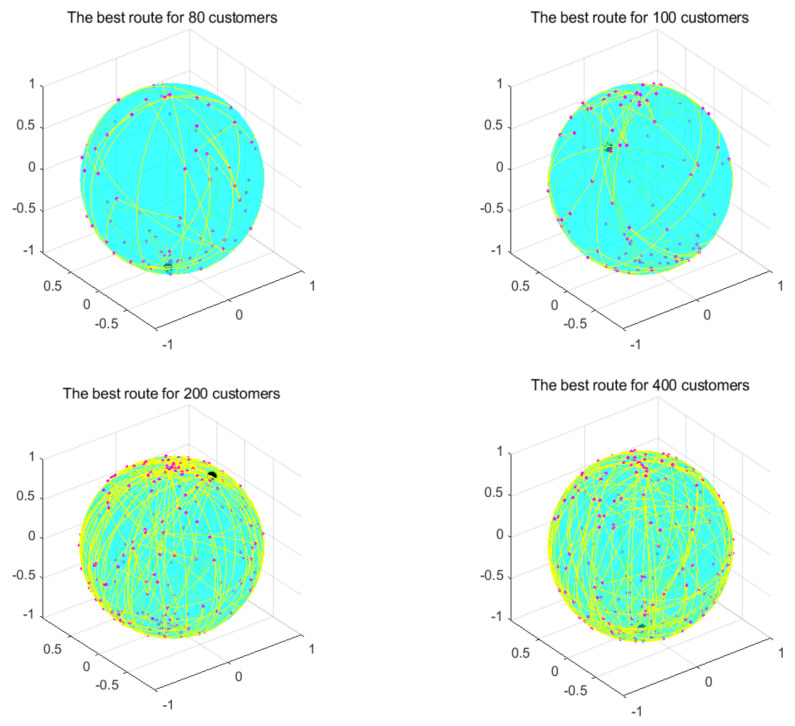
Optimal paths found by the proposed algorithm.

This part tests the performance of the algorithm through four instances of different scales. All algorithms were independently run 30 times to obtain the results, and then the optimal value, the worst value, the average value, and the standard deviation of the result obtained were recorded. As shown in Table 4, the font marked in bold indicates the best value of all algorithms. Figure 10 shows the convergence curve after 300 iterations of the algorithms. Figure 11 shows the ANOVA test of the result after 30 runs. Figure 12 shows the result of 30 runs by algorithms, Figure 13 shows the optimal path found by the proposed algorithm, and Table 5 shows the Wilcoxon rank-sum test, which shows the significance of the difference between the proposed algorithm and other algorithms. It is worth noting that the rank metric in Table 4 was obtained by the Friedman statistical test (Zimmerman et al. [80]), and the fitness values shown in Figure 11, Figure 12, Figure 15, Figure 16, Figure 19 and Figure 20 could be calculated according to the following formula, which represents the distance from the coordinate composed of TD and NV to the coordinate origin. In addition, as the convergence curve of TD is more representative, this paper will mainly analyze the convergence speed of algorithms according to the TD.

$Fitness\;Value=\sqrt{{\left(TD\right)}^{2}+{\left(NV\right)}^{2}}$

It can be seen from the instance with 80 customers that the comprehensive performance of MG-ChOA was better than that of the other algorithms. It obtained the optimal value and the minimum average value, but did not obtain the optimal standard deviation. The convergence curve in Figure 10 displays the excellent convergence ability of the proposed algorithm, which converged to the optimal solution faster than the other algorithms. In addition, GA ranked second overall, and the performance gap between algorithms was very small. The ANOVA test in Figure 11 shows that the result obtained by the proposed algorithm was relatively uniform, which means that it had strong stability and good optimization ability. The running result of Figure 12 shows that the search ability of the proposed method was relatively stable, and results obtained were better than those of other algorithms. Figure 13 shows an optimal path obtained by the proposed algorithm for this instance.

From the instance with 100 customers, we can see that MG-ChOA’s comprehensive performance was superior to that of other algorithms, and it obtained superior average and optimal values to all algorithms. ChOA obtained the optimal standard deviation, which indicated that its performance was stable for this instance. The convergence curve in Figure 10 indicates that the proposed algorithm had excellent convergence ability for the instance of 100 customers. It not only found a better solution, but also had the fastest convergence speed. GA ranked second overall, and the solution searched by GA was only second to the first. The ANOVA analysis in Figure 11 shows that the comprehensive performance of the proposed algorithm was relatively good for this instance, and it had stable search capability. The running result of Figure 12 shows that results obtained by the algorithm were generally better than those of the other algorithms. Figure 13 shows the excellent solution for the proposed algorithm for this instance.

The comparison result of the instance with 200 customers indicated that the proposed algorithm had the strongest capability, and not only was the minimum average value obtained, but also the search result was the best among all algorithms. GA ranked first overall—its performance was more stable than that of the proposed algorithm—and PSO achieved the best standard deviation. The convergence curve in Figure 10 shows that the convergence ability of the proposed algorithm was faster and better than that of the other algorithms for this instance. The ANOVA analysis in Figure 11 shows that, although the search ability of the proposed method was not stable, it could find better solutions than the other algorithms. The running result in Figure 12 shows that results obtained by the algorithm were generally better than those of the other algorithms, and the gap was increasingly obvious. Figure 13 shows a good path obtained by the proposed algorithm for this instance.

The comparison result of the instance with 400 customers indicates the superior ability of the proposed algorithm in this instance, and GWO achieved the best standard deviation; in addition, RPSO ranked second overall. Compared with small-scale instances, except for the genetic algorithm, the other algorithms were relatively stable for this instance, but their search ability was greatly reduced. The proposed algorithm and GA both hadbetter search ability and they found better solutions, but the proposed algorithm had better convergence ability and could find good solutions faster than GA. The ANOVA analysis in Figure 11 indicates that the proposed algorithm’s performance was very strong, although it was not as stable as other algorithms. It had the best optimization ability, and the gap between GA and other algorithms is getting smaller and smaller. Figure 13 shows a feasible route found by the proposed algorithm for this instance.

To sum up, the comparison result above shows that there was little difference in the capability of the algorithms for small-scale instances. When the number of customers increased, the performance of the algorithm started to show a significant difference. Compared with small-scale instances, differences between algorithms for large-scale instances increased significantly. The search ability of the proposed algorithm was still excellent for large-scale instances, and it had fast convergence and search capability. The performance of GA ranked second in the instance above, which was related to the strong search ability of its genetic operators. Both ChOA and GWO are stable because they have adaptive factors. With the increase in the instance’s size, the search ability of other algorithms became weaker and weaker, and the gap between the genetic algorithm and other algorithms became smaller and smaller. In general, the proposed algorithm performed best in the four instances, but the stability of the algorithm needs to be improved, which is related to the richness of improved methods. Finally, Table 5 shows the significant difference between the proposed algorithm and other algorithms in each instance, with a significance level of 0.05. This means that the difference between two groups of data is significant if the *p* value is less than 0.05. Table 5 shows that the experimental results between the proposed algorithm and other algorithms were significantly different in low-dimensional instances. Therefore, the proposed method is better than other algorithms.

### 5.4. Performance Comparison of Algorithms for High-Dimensional Instances

**Figure 14:**
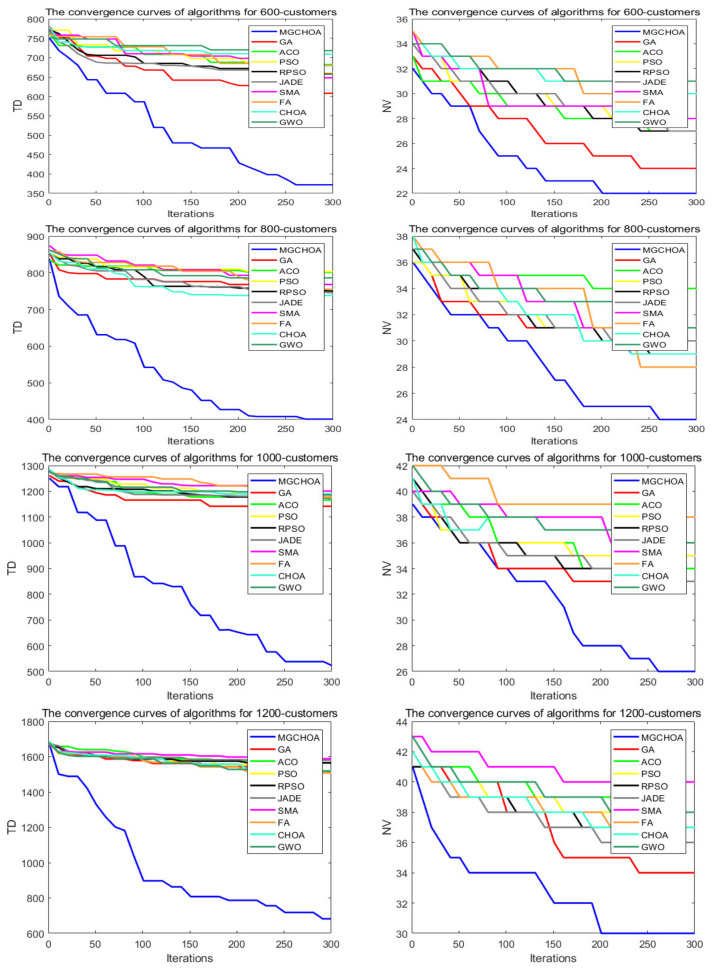
The convergence curves after 300 iterations of algorithms.

**Figure 15:**
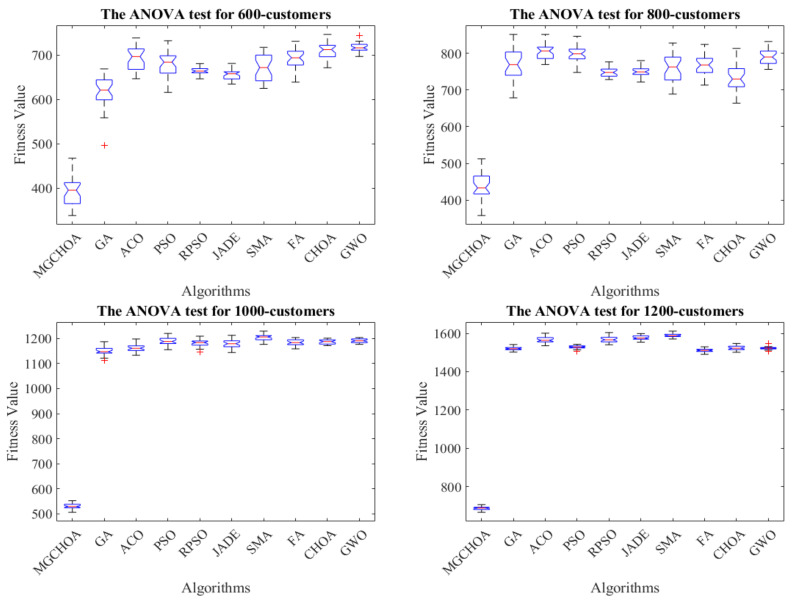
The ANOVA tests of the result after 30 runs, the blue box denotes the data distribution, the red line represents the median value, and the red symbol denotes the abnormal value.

**Figure 16:**
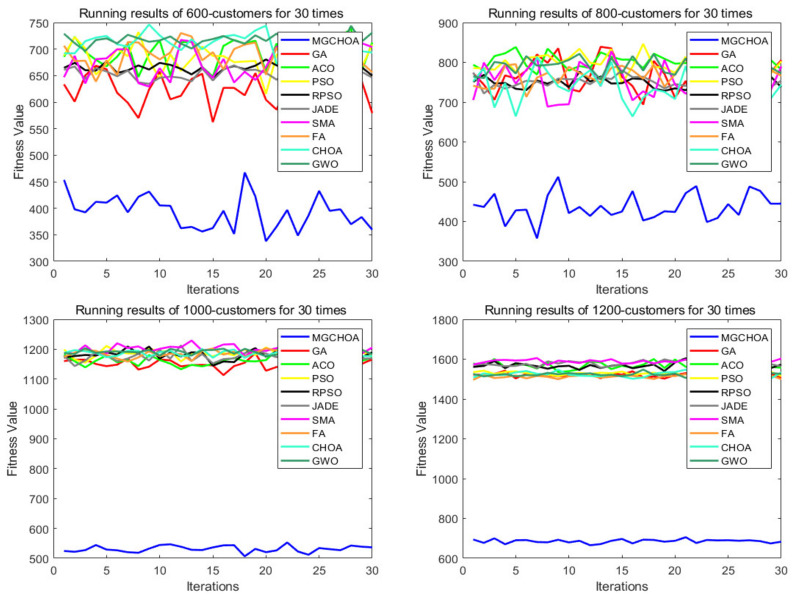
The results of 30 algorithm runs.

**Figure 17:**
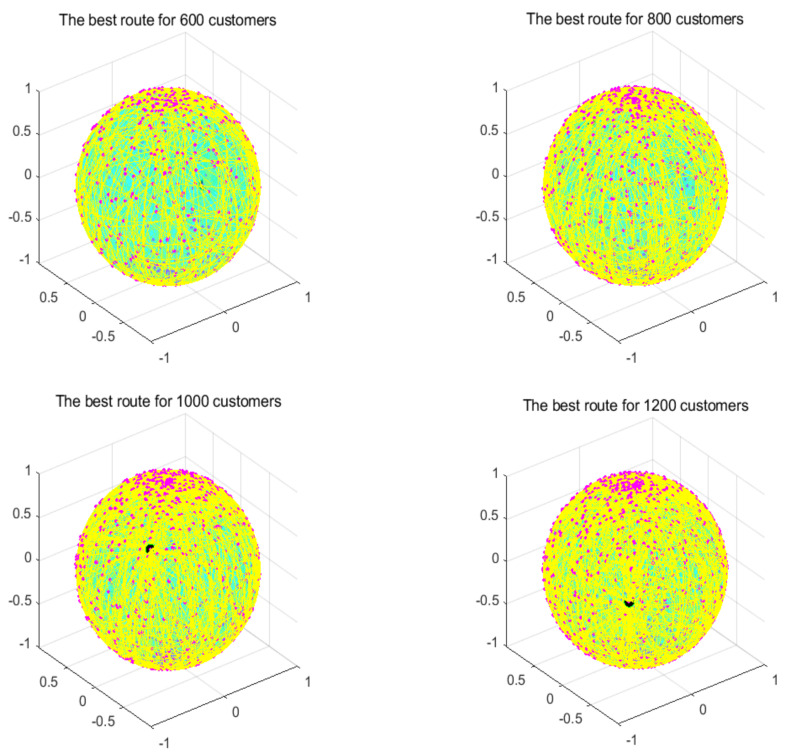
Optimal paths found by the proposed algorithm.

In this section, the search ability of the algorithms is tested through four instances of different scales, namely, instances with 600, 800, 1000, and 1200 customers. All algorithms were independently run 30 times. Table 6 shows the best value, the worst value, the mean value, and the standard deviation of the result obtained, and the font marked in bold represents the optimal value obtained by all algorithms. Figure 14 compares the convergence curve of the algorithm after iterating for 300 generations. Figure 15 shows the result of the ANOVA analysis of the algorithm after 30 runs, Figure 16 displays the result of these algorithms for 30 runs, Figure 17 shows the optimal path of the proposed algorithm, and Table 7 displays the Wilcoxon rank-sum test, which shows the significance of the difference between the proposed algorithm and other algorithms.

From the instance with 600 customers, we can see that MG-ChOA had a better search ability than the other algorithms. It obtained the best value and the optimal average value, and RPSO obtained the best standard deviation; in addition, RPSO ranked second. RPSO and JADE also performed well and had little difference from each other. ACO, ChOA, and GWO had relatively poor search ability. The convergence curve in Figure 14 shows that the proposed method achieved faster convergence than the other algorithms. The ANOVA analysis in Figure 15 shows that the search ability of the proposed method was relatively stable in all algorithms, and the solutions found were better than those of other methods. The running result in Figure 16 shows that, although the search ability of the proposed algorithm fluctuated greatly, the result obtained was generally better than that of other algorithms. Figure 17 shows an excellent solution for the proposed algorithm for this instance.

From the instance with 800 customers, we can see that MG-ChOA obtained the optimal and superior average values to all algorithms, and its comprehensive performance was the best; moreover, RPSO ranked second overall, and JADE obtained the best standard deviation. The convergence curve in Figure 14 indicates that, for this large instance, the excellent convergence speed of the proposed method enabled the algorithm to constantly search for better solutions; in addition, the convergence speed of the proposed method was much faster than that of other algorithms and the solution found was better. Secondly, the search ability of the chimp optimization algorithm was better than that of the GA, which indicates that its adaptive factor well-balanced the search process of the algorithm. The ANOVA test in Figure 15 shows that the search ability of the proposed method was relatively stable for this instance, and the JADE’s performance was the most stable. The running result in Figure 16 shows that results obtained by the proposed algorithm were much better than those of other algorithms, but the performance of most algorithms was unstable. Figure 17 shows an excellent solution found by the proposed algorithm for this instance.

The comparison result of the instance with 1000 customers indicates that the proposed algorithm had the best search ability. It not only found the lowest average value, but also obtained the best result among all algorithms, and the GA ranked second overall. The convergence curve in Figure 14 shows that the proposed algorithm had the best convergence ability for this instance, while the convergence ability of other algorithms became slower and slower; this indicates that the proposed algorithm was still effective for large instances. The ANOVA analysis in Figure 15 reveals that all algorithms were stable, and the proposed algorithm found better solutions than the other algorithms. The running result in Figure 16 shows that the result obtained by the proposed algorithm was generally better than that obtained by the other techniques, and the performance gap between them was obvious. Finally, Figure 17 shows a feasible path obtained by the proposed algorithm for this instance.

The comparison result of the instance with 1200 customers indicates the superior ability of the proposed algorithm for this instance. The performance of other algorithms was relatively stable for this instance, and their search ability was greatly weakened compared with that for small-scale instances. Compared with other algorithms, the proposed algorithm still had a good convergence ability and obvious advantages, and it could find excellent solutions faster. The analysis of the ANOVA test in Figure 15 indicates that these algorithms were relatively stable with little difference from each other. The proposed algorithm still had the best performance, and the gap with other algorithms was the largest. Figure 17 shows a good solution obtained by the algorithm for this instance.

To sum up, the above comparison result shows that the differences in the search ability of these algorithms for large-scale instances became more and more obvious, but the performance of the proposed algorithm was still excellent, with fast convergence ability and good search ability, and the gap was about 30% higher than that for instances with 80 customers. Therefore, this shows that the adaptive factor of the algorithm well-balanced the search ability, and the combination of genetic operators and local search strengthened the exploration and convergence ability of the algorithm; in addition, the multiple-population strategy strengthened the communication between populations, allowing the algorithm to find better solutions faster. Secondly, the performance of the GA generally ranked second, which indicates that its genetic operator had strong search ability. The adaptive factor of the chimp optimization algorithm could well-balance the exploration and exploitation. Therefore, these two methods strengthened the performance and stability of the algorithm. With the increase in the instance’s size, the performance of these algorithms became weaker and weaker, and the gap between other algorithms became smaller and smaller. In general, the proposed algorithm performed best for the above eight instances, but the stability of the proposed method needed to be improved, indicating that the improvement method for the proposed method was successful and efficient. Table 7 shows that the experimental results between the proposed algorithm and other algorithms were significantly different for high-dimensional instances. Therefore, the proposed method was obviously better than other algorithms.

### 5.5. Performance Limit Test of MG-ChOA

**Figure 18:**
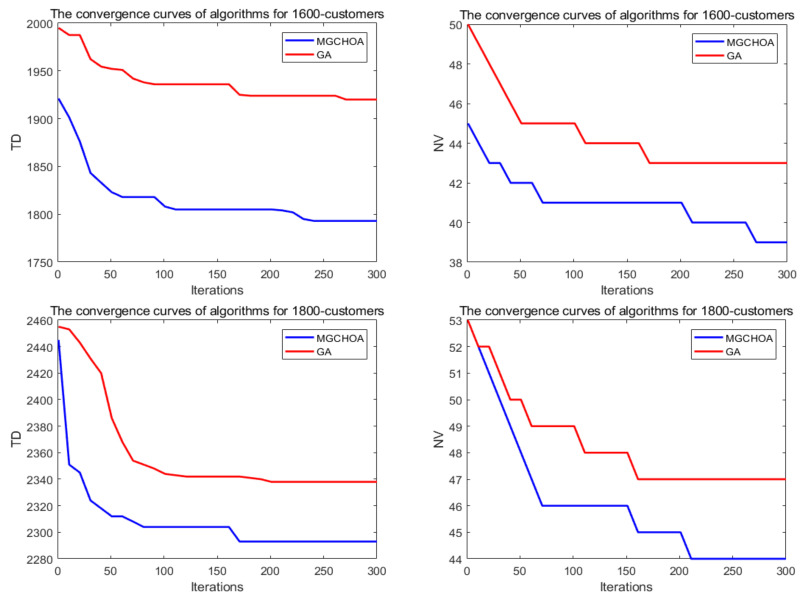
The convergence curves after 300 iterations of the algorithms.

**Figure 19:**
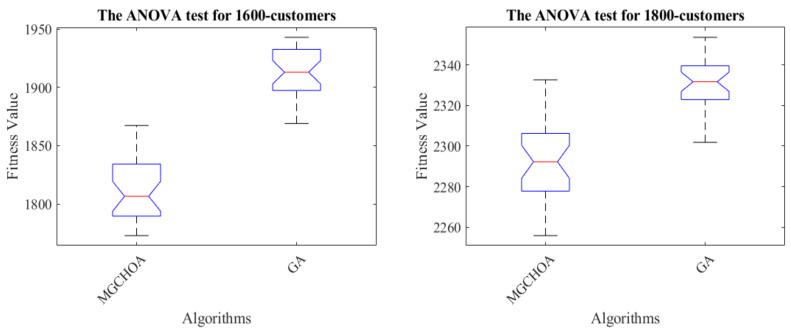
The ANOVA tests of the results after 30 runs, the blue box denotes the data distribution, the red line represents the median value, and the red symbol denotes the abnormal value.

**Figure 20:**
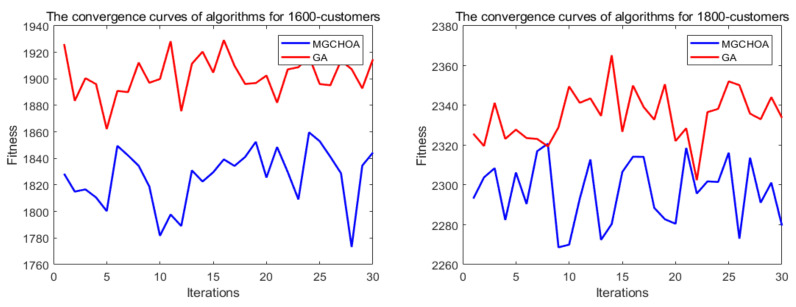
The results of 30 runs of the algorithms, the blue box denotes the data distribution, the red line represents the median value, and the red symbol denotes the abnormal value.

**Figure 21:**
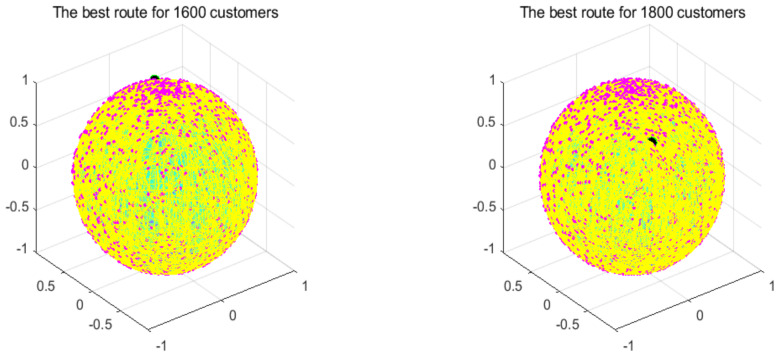
Optimal paths found by the proposed algorithm.

In order to measure the boundaries of the algorithm proposed in this paper, this section adds two additional instances to verify the limits of the algorithm, which are instances of 1600 customers and 1800 customers. Table 8 records the best value, the worst value, the average value, and the standard deviation of the algorithm for these instances. Next, this section uses the GA for comparison with the proposed algorithm in terms of their convergence speeds. Figure 18 shows the convergence speed of the algorithm. Figure 19 and Figure 20 respectively show the ANOVA test and the result statistics of the algorithms when run 30 times. To sum up, the convergence speed of the algorithm became slower and slower. Compared with the 1200-customer instance, the 1600-customer instance had a significant decline, and the convergence ability of the proposed algorithm on the 1800-customer instance was close to the limit. Moreover, the analysis of the ANOVA test of the algorithm showed that its stability became weaker and weaker. Therefore, there is still much more room to improve the performance of the algorithm. Table 9 shows that the results of these two algorithms were significantly different. Figure 21 shows an excellent solution for the proposed algorithm for these instances

### 5.6. Performance Analysis of Different Strategies

**Figure 22:**
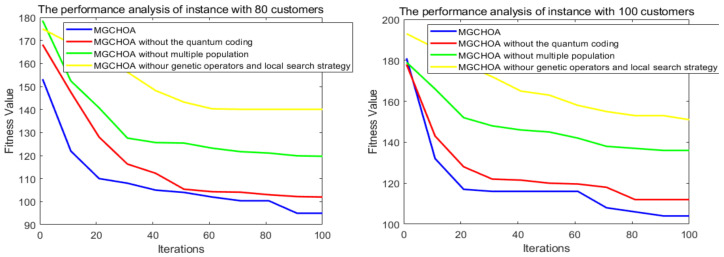
The results of various improved methods on the 80-customer and 100-customer instances.

This section analyzes the impacts of different improved methods on the overall performance of the proposed algorithm to further test the effectiveness of these improved methods. Figure 22 shows the search results of various improved methods on the 80-customer and 100-customer instances. In this paper, we propose initializing the population by using quantum coding. The main idea is as follows. Firstly, the population generated by the quantum coding technique is twice as large as the original population, which increases the diversity of the initial population. Secondly, this method mainly includes two trigonometric functions, which generate individuals with good probability distribution, and this method well-balances the initial exploration and exploitation of the algorithm. As can be seen from Figure 22, the method of initializing the population by quantum coding sped up the convergence of the algorithm and caused it to find a feasible solution faster, but the effect was not obvious.

Moreover, genetic operators and the local search strategy were introduced into the algorithm. Genetic operators have strong search ability and can broaden the search scope of an algorithm. Their performance is very suitable for solving path problems, while local search algorithms can find better solutions in the neighborhoods of these excellent solutions found in each iteration. Therefore, the combination of the above two strategies can greatly improve the exploitation and exploration ability of algorithms and help them to find solutions with high accuracy. Figure 22 shows that this method made the largest proportion of contributions to the performance of the proposed algorithm, which shows that this method was suitable for this algorithm and enhanced the effectiveness of the proposed algorithm. This paper also introduced a multiple-population strategy and generated two populations. This method achieved communication between populations through migration operators, so the algorithm could find multiple excellent solutions in each generation, which enhanced the exploration ability. Figure 22 shows that the introduction of this method significantly improved the convergence speed of the proposed method, indicating that the proposed method could be feasibly integrated with multiple-population strategies. In addition, this study also conducted experiments to select the best population number, and the results are shown in Table 10, where p is the population’s number. Table 10 shows that the optimal result was obtained when the population number was set to 2, so the population number of the method proposed in this paper was 2.

## 6. Conclusions and Future Work

The chimp optimization algorithm (ChOA) is a new swarm intelligence algorithm that has excellent search ability and is suitable for solving continuous problems. The characteristic of this algorithm is that the convergence speed is fast during the initial stage of iteration, and the solution’s accuracy is high, but the convergence ability is weakened during the later stage of iteration, and it easily falls into the local optimum. Furthermore, the algorithm can adaptively adjust its exploration and exploitation when searching the solution space because the algorithm has well-designed adaptive factors to balance the exploitation and exploration in the process of optimization.

Although the performance of the chimp optimization algorithm itself was superior, it was not suitable for dealing with discrete optimization problems in real life, and the convergence speed of the algorithm became slower and slower, so it easily fell into the local optimum. Therefore, this paper improved the performance of the algorithm according to the shortcomings above, and the multiple-population strategy, genetic operators, and local search strategy were integrated into the algorithm to enhance the overall exploration ability and convergence speed of the proposed method. The multiple-population strategy initializes multiple populations, uses migration operators to exchange information among various populations, and finally selects excellent individuals to enter the next generation through manual selection operators. The combination of genetic operators and local search strategy not only strengthened the overall search ability of the algorithm, but also improved the convergence speed so that the algorithm could find better solutions faster.

In order to verify the effectiveness of the algorithm’s improvement, this paper analyzed the performance of the proposed algorithm and several excellent algorithms for instances of different scales. The test results indicated that the proposed method was effective and superior in solving the spherical VRPTW model, and its results were better than those of other algorithms. With the increase in the instance’s size, the gap became more obvious. Finally, this paper analyzed the improvement method for this method, and the experimental result showed that the improvement of the proposed algorithm was effective.

However, according to the NFL theorem, the proposed algorithm still has some limitations, such as the search ability of the algorithm not being stable enough and the running time being relatively long. Therefore, the performance of MG-ChOA will continue to be explored and improved through practical applications in the future, and the spherical VRPTW model studied in this paper will also be discussed and studied in combination with green logistics, robot path planning, and other topics.

## Figures and Tables

**Table 1:** Notations and their descriptions.

N	Number of customer nodes
C	Set of customers as {0,1,…*N*}, where 0 is the depot
V	Set of vehicles, V = {1,…,*K*}
K	Number of vehicles
k	Index of vehicles (k∈V)
$d_{ij}$	Distance between nodes *i* and *j*
$t_{ij}$	Travel time between nodes *i* and *j*
$ET_{i}$	Earliest arrival time at node *i*
$LT_{i}$	Latest arrival time at node *i*
$ST_{i}$	Service time of node *i*
$q_{i}$	Demand of customer *i*
$Q_{k}$	Capacity of vehicle *k*
$T_{ik}$	Arrival time of vehicle *k* to *i*
$w_{ik}$	Waiting time at customer *i*

**Table 2:** The control parameter settings of each algorithm.

Algorithms	Authors	Parameters
MG-ChOA	This paper	*P* = 2, *m* was calculated by Gaussian mapping, *pc* = 0.9, *pm* = 0.1, and the number of customers deleted in the local search strategy was 15% of the total
GA	Holland [20]	*pc* = 0.8, *pm* = 0.8
ACO	This paper	The pheromone was set to 4, heuristic information was 5, waiting time was 2, time window width was 3, parameter controlling ant movement was 0.5, evaporation rate of pheromone was 0.85, and the constant affecting pheromone updating was 5
PSO	Kennedy et al. [32]	The inertia weight was 0.2, global learning coefficient was 1, and the self-learning coefficient was 0.7
RPSO	Borowska [72]	The inertia weight was 0.6, acceleration constants were c1 = c2 = 1.7, and the number of particles with the worst fitness *p* was set as 3.
JADE	Borowska [73]	Parameters of the algorithm changed adaptively
SMA	Li et al. [79]	The parameter controlling foraging was 0.03
FA	Yang [35]	The basic value of the attraction coefficient was 0.8, the mutation coefficient was 0.8, and the light absorption coefficient was 0.8
ChOA	Khishe et al. [25]	Parameters of the algorithm changed adaptively
GWO	Mirjalili et al. [37]	Parameters of the algorithm changed adaptively

**Table 3:** The comparison of the results for four Solomon datasets.

Datasets	Best Known			MG-ChOA		%Difference in TD
	NV	Authors	TD	NV	TD	
C101	Rochat	10	828.94	10	828.94	0.00
R102	Rochat	17	1486.12	18	1473.62	−0.84
R201	Homberger	4	1252.37	9	1165.10	−7.49
RC105	Berger	13	1629.44	8	1234.1	−3.20

**Table 4:** Experimental results of algorithms for instances of 80, 100, 200, and 400.

Instances	Algorithms	Best	Worst	Mean	Std	Rank
80	MG-ChOA	**74.6915**	**89.1348**	**82.1517**	3.5346	1
	GA	77.8541	101.4105	91.6031	4.7625	2
	ACO	94.4498	121.3766	107.9738	6.2796	7
	PSO	105.8825	117.5272	113.3724	3.1874	6
	RPSO	92.8541	116.4105	106.8345	4.9541	3
	JADE	93.0822	115.5015	107.3124	6.6533	5
	SMA	112.3210	126.2165	118.7991	3.4966	10
	FA	113.1794	125.5953	117.9425	**2.7192**	8
	ChOA	107.4037	123.4656	115.5410	3.8180	9
	GWO	105.7460	118.7477	112.6344	3.0227	4
100	MG-ChOA	**85.2594**	**109.3002**	**97.0352**	5.8962	1
	GA	102.7593	133.5235	114.7344	7.3826	2
	ACO	153.0400	176.0259	162.3654	5.7970	9
	PSO	152.1939	168.1939	160.8924	3.6269	7
	RPSO	132.9432	159.1628	149.4894	6.7970	6
	JADE	120.0249	157.4222	139.4843	8.3004	5
	SMA	160.3001	174.0341	165.4979	3.8072	10
	FA	158.1066	171.3606	165.7973	2.9858	8
	ChOA	147.7598	157.5543	151.6967	**2.5188**	4
	GWO	144.5013	156.8961	151.5078	3.4927	3
200	MG-ChOA	**92.4894**	**171.5740**	**130.6591**	16.0142	2
	GA	185.4160	214.2204	199.0548	7.6380	1
	ACO	269.0036	308.7602	284.6381	8.3779	5
	PSO	283.7265	305.1323	292.6501	**4.4139**	4
	RPSO	236.7104	282.2716	258.7564	10.2254	3
	JADE	237.7484	293.4480	261.7672	10.8409	6
	SMA	287.7402	323.2023	309.9585	7.7234	7
	FA	285.0593	329.6599	307.5770	9.8884	8
	ChOA	294.4387	326.0060	310.2144	8.7833	9
	GWO	296.0258	328.0148	315.6643	7.8676	10
400	MG-ChOA	**248.9622**	**319.9879**	**276.4939**	18.1938	1
	GA	366.3048	433.7308	401.4895	16.6535	3
	ACO	454.0676	498.4261	482.8094	9.0784	6
	PSO	451.2690	502.3332	476.5840	12.6521	7
	RPSO	443.9548	480.0339	461.6864	8.34601	2
	JADE	433.6780	480.9550	460.5177	10.7607	4
	SMA	454.1847	502.8520	477.1359	12.5691	9
	FA	471.2963	518.0317	492.6073	12.2617	10
	ChOA	461.8166	493.2990	475.5718	9.2174	5
	GWO	483.0315	502.0965	492.3936	**4.2985**	8

**Table 5:** The results of Wilcoxon rank-sum test results for low-dimensional instances.

Instances	GA	ACO	PSO	RPSO	JADE	SMA	FA	ChOA	GWO
80	1.73 × 10^−6^	1.73 × 10^−6^	1.73 × 10^−6^	1.73 × 10^−6^	1.73 × 10^−6^	1.73 × 10^−6^	1.73 × 10^−6^	1.73 × 10^−6^	1.73 × 10^−6^
100	1.92 × 10^−6^	1.73 × 10^−6^	1.73 × 10^−6^	1.73 × 10^−6^	1.73 × 10^−6^	1.73 × 10^−6^	1.73 × 10^−6^	1.73 × 10^−6^	1.73 × 10^−6^
200	1.73 × 10^−6^	1.73 × 10^−6^	1.73 × 10^−6^	1.73 × 10^−6^	1.73 × 10^−6^	1.73 × 10^−6^	1.73 × 10^−6^	1.73 × 10^−6^	1.73 × 10^−6^
400	1.73 × 10^−6^	1.73 × 10^−6^	1.73 × 10^−6^	1.73 × 10^−6^	1.73 × 10^−6^	1.73 × 10^−6^	1.73 × 10^−6^	1.73 × 10^−6^	1.73 × 10^−6^

**Table 6:** Experimental results of the algorithms for instances of 600, 800, 1000, and 1200.

Instances	Algorithms	Best	Worst	Mean	Std	Rank
600	MG-ChOA	**337.9706**	**467.3819**	**393.3942**	31.1964	1
	GA	496.1133	668.4894	615.0010	37.3412	4
	ACO	646.3231	738.3302	692.2071	26.8871	8
	PSO	615.6234	732.0994	679.0999	25.8592	5
	RPSO	646.1737	680.3887	663.5755	**8.2341**	2
	JADE	634.4642	680.8589	654.7950	11.2532	3
	SMA	624.6327	717.2041	670.4092	28.6820	6
	FA	638.7109	730.8624	689.8933	23.4781	7
	ChOA	671.0393	746.3261	709.2050	18.7072	10
	GWO	696.4319	743.7724	716.6254	9.9470	9
800	MG-ChOA	**357.7428**	**512.2769**	**436.7981**	32.8699	1
	GA	678.5272	851.5621	769.3450	44.8163	8
	ACO	769.4406	851.4780	804.6975	19.5546	10
	PSO	747.8239	846.6075	797.8163	21.5773	9
	RPSO	728.5422	776.7191	748.1957	12.2668	2
	JADE	721.68571	780.1052	750.1521	**12.0138**	3
	SMA	688.8120	827.9618	757.8355	39.4238	6
	FA	713.2352	824.3031	767.2942	24.4921	5
	ChOA	664.0851	813.3595	733.6243	37.8625	4
	GWO	756.3863	832.1562	791.0453	19.8800	7
1000	MG-ChOA	**506.5729**	**552.8227**	**531.1736**	10.5891	1
	GA	1113.3512	1186.7736	1148.6414	14.9046	2
	ACO	1132.8896	1197.7980	1161.3229	13.9392	3
	PSO	1154.9312	1219.9879	1188.6006	15.3536	9
	RPSO	1145.0236	1209.0242	1182.4126	15.3321	6
	JADE	1143.6047	1212.5595	1177.5565	15.4292	8
	SMA	1176.3676	1229.0331	1203.5824	12.5634	10
	FA	1158.5719	1203.2321	1183.8478	11.3573	5
	ChOA	1171.0874	1200.9404	1185.5053	9.2586	4
	GWO	1176.2921	1203.2903	1190.6301	**8.3314**	7
1200	MG-ChOA	**665.4418**	**705.5305**	**685.8351**	9.0505	1
	GA	1502.4781	1542.2096	1519.9860	9.4525	3
	ACO	1535.6057	1601.0904	1567.2093	16.7193	7
	PSO	1505.9321	1543.2443	1528.1268	9.0864	5
	RPSO	1540.4621	1604.3669	1568.7338	14.3821	9
	JADE	1554.5170	1598.9232	1577.1516	11.8324	8
	SMA	1570.9568	1611.9386	1589.1695	9.2840	10
	FA	1490.7260	1530.2589	1510.9637	8.8396	2
	ChOA	1501.5574	1547.4414	1523.5250	11.8448	6
	GWO	1505.9274	1547.6684	1522.6388	**7.6334**	4

**Table 7:** The Wilcoxon rank-sum test results for high-dimensional instances.

Instances	GA	ACO	PSO	RPSO	JADE	SMA	FA	ChOA	GWO
600	1.73 × 10^−6^	1.73 × 10^−6^	1.73 × 10^−6^	1.73 × 10^−6^	1.73 × 10^−6^	1.73 × 10^−6^	1.73 × 10^−6^	1.73 × 10^−6^	1.73 × 10^−6^
800	1.73 × 10^−6^	1.73 × 10^−6^	1.73 × 10^−6^	1.73 × 10^−6^	1.73 × 10^−6^	1.73 × 10^−6^	1.73 × 10^−6^	1.73 × 10^−6^	1.73 × 10^−6^
1000	1.73 × 10^−6^	1.73 × 10^−6^	1.73 × 10^−6^	1.73 × 10^−6^	1.73 × 10^−6^	1.73 × 10^−6^	1.73 × 10^−6^	1.73 × 10^−6^	1.73 × 10^−6^
1200	1.73 × 10^−6^	1.73 × 10^−6^	1.73 × 10^−6^	1.73 × 10^−6^	1.73 × 10^−6^	1.73 × 10^−6^	1.73 × 10^−6^	1.73 × 10^−6^	1.73 × 10^−6^

**Table 8:** Experimental results of algorithms on instances of 1600 and 1800 customers.

Instances	Algorithms	Best	Worst	Mean	Std	Rank
1600	MG-ChOA	**1772.9074**	**1867.3117**	**1811.1547**	25.8704	1
	GA	1869.0448	1942.9288	1913.8718	**19.4836**	2
1800	MG-ChOA	**2255.8563**	**2332.6466**	**2291.6476**	20.7723	1
	GA	2301.8192	2353.5944	2330.8980	**13.3407**	2

**Table 9:** The Wilcoxon rank-sum test results.

Instances	GA
1600	1.73 × 10^−6^
1800	1.73 × 10^−6^

**Table 10:** Effects of different population numbers on the result.

Instances	P = 1	P = 2	P = 3	P = 5	P = 6	P = 7	P = 8
80	82.4214	74.6215	83.5353	83.3345	84.6859	86.2861	85.4101
200	124.7481	92.1894	128.2701	137.8198	153.1216	158.3709	171.8740
600	351.8525	337.4706	364.4159	392.7295	410.9527	421.8533	453.388
800	402.7831	387.2349	424.9258	443.1142	465.9428	477.8761	512.2769

## Data Availability

Not applicable.
